# Physiology

**Published:** 1825-04-01

**Authors:** 


### Quarterly Periscope

OF

### PRACTICAL MEDICINE;

BEING

### The Spirit of the Medical Journals,

*Foreign and Domestic;*

WITH COMMENTARIES.

I.

PHYSIOLOGY.

——— latas à numine leges
Religiosa doce, mentesque cupidine veri
Allice.

1. *On Vital Power, or Individual Idiosyncrasy.** M. Martinet justly observes, that too much importance is attached to *post-mortem* appearances, and too little account is taken of individual idiosyncrasy. Thus, we see some people die, and yet the organic lesion found, on dissection, is not at all calculated to account for the fatal event. By vital power, or *resistance,* (as Martinet terms it) our author does not mean the energy of functions, nor the excess of irritability or activity in a given individual—nor yet that harmonious balance of the different systems of the body, which is designated a *good constitution.* By vital resistance, he means that power, by virtue of which a mân supports, without much inconvenience, the action of those causes which have a tendency to destroy him—a power which may exist in the highest degree in individuals of the weakest, as well as in those of apparently the strongest constitutions—in those who are irritable, as well as in those who are placable. In the difference of degree, then, of this vital power of resistance, we may find some clew for reconciling certain contrarieties of opinion that divide the medical world. It may tend to bring into accordance those physicians who maintain that, in fevers, death is the result of a local lesion, and those who, on the other hand, believe that death does not depend on the actual state of particular organs (which

* Sur les differens Degrés de Resistance Vitale dans les Maladies, deduites des rapports des lesions organiques, avec leurs effêts. Par L. Martinet, M.D. REVUE MEDICALE, OCTOBRE, 1824.

often present no trace of disease)—and maintain that we cannot regard a slight redness of the cerebral meninges, of the pleura, or of the stomach, as the sole cause of the fatal event, when we every day see much greater lesions of structure, in other individuals, attended with comparatively trifling disturbance of function. In order to throw some light on this subject, let us advert to certain phenomena which are constantly presenting themselves to our senses. Let us take *sensibility*, for example, as manifested in the cutaneous surface. In one person, the prick of a needle or other sharp instrument will produce but a slight and momentary pain—in another, exquisite suffering—in a third, syncope—in a fourth, tetanus;—yet, in all these, the local effects will be precisely the same; namely, a slight inflammatory areola round the puncture. Whence can proceed these various effects, but from diversity of individual disposition—in other words, idiosyncrasy?

If, from the cutaneous sensibility, we pass on to the propensities or aptitudes to contract such and such diseases, we shall find an infinite diversity in individuals. Some people will be exposed to a focus of infection, for days and weeks, with impunity; while others, of apparently similar constitutions, soon fall victims to the deleterious miasms. It is not robustness of constitution, nor equanimity of mind which resists the contagion—but often the very reverse. And this is not only the case with small-pox, typhus, and plague—the same observations will apply to diseases which are caused or propagated in other and different modes. Every day we see blows on the head—suppressions of perspiration by cold—excesses in eating or drinking, borne with little or no sensible effects, by some people—while, in others, the slightest concussion—the least excesses in exercise, eating, or drinking, will be followed by violent inflammations of the head, chest, or digestive apparatus. What, but idiosyncrasy, can account for these differences of result? Again, let us look at these individuals when actually affected with diseases. Some get well in a few days—others linger a long time under the same affection, and a third party are quickly cut off and perish—the malady appearing of the same nature in all. Nor is it the intensity of the disease, or the extent of the inflammation, which can explain these differences. Take pneumonia, for example:—in some people, who die, a small portion only of one lobe will be inflamed—in others, almost the whole of both lobes will be phlogosed, and yet they will speedily recover. In these last, the vital resistance is superior to the disease—in the former class, it is inferior. Now, as it is quite impossible to say, in the beginning of a disease, whether or not the degree of vital power is equal to overcome the malady, since there are no marks of external form or internal function by which it can be known—so the science of prognosis is proverbially fallible, and, it is to be feared, will ever remain so! A history of the individual's habits, former ailments, and peculiarities of disposition, is the only document on which we can place any confidence—but how imperfectly is this investigated by the routine practitioner, and the fashionable physician!

ILLUSTRATIONS.

*Case* 1. Tellier, 32 years of age, of robust constitution, and athletic frame, and who had previously enjoyed good health, was affected a fortnight ago, with a diarrhœa, which only lasted 24 hours, and ceased without medicine. From this time, he was in good health, till the night of the 5th of March, 1824, when he was seized with violent cholic and diarrhœa. *6th.* Though feeble and fatigued, he went out: but finding himself unable to work before breakfast, he took a small quantity of brandy, at seven o'clock in the morning. He had scarcely swallowed it, when he became faint, and was obliged to retire to bed. Vomiting and purging now came on which lasted all that day and succeeding night. The matters ejected, both by vomiting and purging, were colourless, but accompanied by acute pains in the abdomen, anxiety, and restlessness. By the advice of a physician, the patient confined himself to the use of lemonade (a curious prescription for vomiting, griping, and purging!) On the morning of the 7th, he was carried to the Hotel Dieu. The vomiting and purging had nearly ceased, but what passed was of a green colour. Carefully examined, he now presented the following symptoms:—great prostration of strength—lies on his back—eyes hollow and fixed—face pale, and features greatly changed—answers with difficulty to questions—complains of pain only in his throat—but there was tenderness on pressure at the epigastrium—thirst—frequent eructation—vomiting every quarter of an hour, of a greenish fluid—tongue coated yellow—abdomen tense—pulse imperceptible at the wrist, but the stethoscope made the cardiac pulsations 80 in the minute—skin dry—heat natural on the trunk, but the extremities cold. In this state, he lingered out till ten o'clock in the evening of the 8th.

*Dissection.* The vessels of the meninges of the brain slightly injected—brain itself natural—lungs sound and crepitous—nothing wrong in this quarter. *Abdomen.* Stomach pale externally—the pyloric half of the mucous membrane exhibited a rosy tint, when washed, but no other alteration—duodenum empty, and its mucous lining of a yellowish colour—no alteration in the upper portion of the small intestines, but, towards their middle, they exhibited a violet tinge for nearly two feet in extent, externally, while the corresponding portion of mucous membrane was of a vivid red colour, particularly about the valvulæ conniventes—lower portion of ileum contracted, and its mucous membrane inflamed, with streaks of white, apparently produced by a purulent infiltration into the sub-mucous cellular tissue. The large intestines, the liver, and other abdominal organs were in a natural condition.

*Remarks.* It is quite evident that the quantum of inflammation found in this case, bears no proportion to the violence of the symptoms, and the suddenness of the fatality—especially when we find the patient in the prime of life, and of an excellent constitution. The case proves that, notwithstanding all this, the disturbance induced in the vital functions by so slight a phlogosis, was yet greater than the vital power of resistance, and consequently death was the result. From this and other cases, we may reasonably infer that, in certain constitutions or idiosyncrasies, a disorder of a vital part, or parts, may be so slight as scarcely to leave a trace of its existence, *post mortem*, and yet be quite sufficient to destroy the life of the individual. This appears to be exemplified in the following instance.

*Case* 2. A girl, 16 years of age, having been affected with severe cephalalgia for a fortnight, entered the Hotel Dieu, on the 16th December, 1823. On the 17th, she presented the following symptoms;—intellectual functions free—severe pain of head in the vertex and forehead—tongue moist and not red—a little whitish at the base—abdomen not sensible to pressure, and of the natural temperature—respiration free, and the respiratory sound audible in every part of the thorax—pulse natural—lies across the bed. With the exception of this last symptom, which was attributed to whim and the head-ache, the young patient presented no tangible symptom of disease. Some opening medicine was prescribed by M. Recamier, with pediluvia. 18th. Same state—no fever—head-ache continues. Fifteen leeches behind the ears—pediluvia—lemonade. 19th. The epigastrium a little sensible to pressure—an expression of stupor about the countenance—the pulse a little accelerated and full—head-ache the same—tongue white, but moist. In the evening, a *smart fever* rose, and continued for some hours, when the patient suddenly expired.

*Dissection.* Body rather embonpoint—tunica arachnoidea transparent and perfectly natural—no injection of the pia mater—no congestion of vessels—only a few drops of fluid in the ventricles—no traces of alteration in the brain, cerebellum, or medulla oblongata—lungs crepitous and sound—in short, there was no vestige of disease in any part of the body.

*Remarks.* It cannot be said that this girl died of inflammation of the brain, its membranes, or any other structure in the body. Yet fever was unequivocally established before death. The head appears to have been the seat of the disease; but there is no proof of actual phlogosis, at least, in the vascular system of the parts. The sensibility merely seems to have been engaged—but the disorder of the sentient system was, in this case, sufficient to destroy life, before any tangible or visible change in the structure of the parts took place. Thus, we see, also, that any excessive affection of the sentient system may, in certain individuals, prove fatal, and that very suddenly, whether the impression be of a painful or of an agreeable nature. Sudden joy, sudden fear, excruciating pain, have quickly extinguished vitality—so frail is the tenure of life!

*Case* 3. A man, 38 years of age, was received into the Hotel Dieu, in January, 1824, complaining of slight pains in his loins, with some looseness of bowels. Lavements—diluents. In the course of a few days, the pains, which had never been accompanied by any pyrexia, disappeared—the patient began to take food, and was considered convalescent. One day he complained, at the medical visit, of general *malaise*, and that his appetite had left him. His pulse and respiration were natural. The physician thought it unnecessary to order him any medicine. In ten minutes afterwards, he appeared in great agitation, and a prey to some severe suffering. His intellectual functions were in perfect integrity. He complained that his belly felt tense and swelled, although it felt soft and natural to the hand of another person—he said he felt his breathing difficult, although the chest sounded well in every direction, and the respiratory murmur was every where heard by means of the stethoscope. The features, however, altered more and more—the pulse acquired frequency—he felt inclination to vomit—he began to cough—he had thirst—and he expectorated five or six ounces of a whitish glairy fluid streaked with blood. Relieved by this expectoration, the patient got up to go to the water-closet, but not being able to discharge any thing, he returned to his bed, without assistance. In a few minutes he expired—not quite an hour from the first invasion of the symptoms above described.

*Dissection.* The brain and its membranes, examined with the greatest care, offered no trace of congestion, no redness, no sensible deviation from that of health. The lungs were perfectly sound and crepitous. A few drachms of clear liquid were seen in each cavity of the pleura—and the same in the pericardium, which, with the heart, was sound in structure. The peritoneum was healthy, but there were full 16 ounces of clear serum in its cavity. The intestinal tube, throughout its whole extent, was colourless—its mucous lining pale. There was no appreciable alteration in any of the other viscera or parts of the body, besides the serous effusion above-mentioned.

The following case will be found still more extraordinary.—

*Case* 4. Rosina Choisi, 20 years of age, of lymphatic temperament, entered the Hotel Dieu, on the 29th of January, 1822, in a state of considerable moral depression, and from whom no satisfactory information could be obtained, although she appeared perfectly sensible of the questions put to her. On examination next day, (30th January) she presented the following phenomena: abdomen tense and painful on pressure—tongue dry, and covered with a black crust, as were the teeth and lips—continual moaning, and complaint of acute pain—inability to reply to questions asked—pulse small, hard, and quick—extreme prostration of strength—breathing free. *Forty leeches to the abdomen—fomentations—emulsions—lavements.* 31st. Tongue not quite so dry—abdomen still very painful, especially on the right side. In other respects, the same. 20 *leeches to the painful parts—semicupia—lavements.*—*Feb.* 1. The tongue is becoming moist and cleaning a little—but the features are sunk and greatly altered—belly still very painful—no alvine evacuation since her entry into the hospital—pulse scarcely perceptible. *Twenty leeches to the abdomen.* 2nd. Tongue again dry—belly still tense, and painful—the pulse is perceptible and very rapid—prostration extreme. *Twenty leeches to the abdomen.* 3rd. diminution of the abdominal pain, but not of the tension and swelling—tongue a little moist —pulse quick and feeble. 4th and 5th. The symptoms rather mitigated than otherwise. 6th. Vomiting has supervened—great agitation and delirium—abdominal pain and fever augmented. *Blisters, semicupia, fomentations, &c.* 7th and 8th. Increase of prostration—constant delirium and restlessness. *Camphor, æther, &c.* 9th. Died this day.

*Dissection.* A most minute examination was made, and not a trace of morbid structure or alteration of any kind could be detected in the brain or its envelopes, in the chest, or in the abdominal viscera.

*Remarks.* This female was treated for peritoneal inflammation, and it cannot be denied that she presented the principal phenomena of that phlogosis. It is possible, too, that such an inflammation did exist, but that the extreme depletion by leeches dissipated it. We are doubtful, indeed, whether depletion was not carried too far in this case, and whether the patient did not die of the typhoid fever accompanying the phlogosis (if it did really exist) together with the immense loss of blood by leeching. Twenty leeches applied to the abdomen, when the pulse was scarcely perceptible at the wrist, was what the phlebotomising Englishman would hardly have ventured on, although he is ridiculed on the Continent for his ultra-depletory operations.

*Case 5.* Marianne, aged 34 years, had been irregular in menstruation from the age of thirteen to twenty-four—during which period she suffered from various anomolous affections. From this time till the age of 33, she was regular and healthy. At the latter epoch, she again became irregular, and her health deranged. The most prominent feature of her complaint was now hæmaturia. The lower extremities became œdematous, but were reduced by bandaging. The hæmaturia diminished, and menstruation again appeared. At this period, a pain fixed itself in the epigastrium, was increased after food—was often accompanied by nausea and vomiting, with occasional dyspnœa. These symptoms increasing, the patient entered L'HOTEL DIEU in the month of April, 1824, where she staid only ten days, still complaining of the abovementioned symptoms. Emetics—leeches—bark—assafœtida—baths, had no good effect, and she left the hospital unrelieved of the complaint, which was unaccompanied by pyrexia. On the 3rd of June, same year, she returned, and now complained of a sense of constriction in the chest, difficulty of breathing, slight head-ache, vertigo, suffusion of the eyes, thirst, inappetency, white tongue, severe pain in epigastrio, on the least pressure, constipation, full pulse, some heat of skin. *Ptisans, lavements, baths.* On the 4th and 5th, the pectoral symptoms were relieved, but she fainted each time she came out of the bath. The epigastric pain continued undiminished. On the 6th, she died in an attack of lypothymia.

*Dissection.* There was a slight serous infiltration between the membranes of the brain. The vessels of the pia mater were injected—the right hemisphere of the brain rather soft. The lungs were sound—the mucous membrane of the bronchia red in some points—heart enlarged, and very soft—left ventricle dilated very much, but without diminution of its parietes. The mucous membrane of the stomach was of a dark brown colour, and softened for a considerable space. The small intestines were red in several places, the mucous membranes corresponding with these places being covered with a whitish, purulent matter. Large intestines sound—liver granulated and very hard—spleen gorged with black blood.

*Reflections.* M. Martinet observes, that the appearances on dissection, in this case, were far from proportionate to the symptoms and suddenly fatal termination. Indeed, we can hardly look upon the disease as a "*gastroenterite*" at all. We think the head and heart were the principal foci of disease, and that the pain in the epigastrium was sympathetic or nervous, rather than inflammatory. The softening of one hemisphere of the brain was a formidable item.

*Case* 6. A man, 36 years of age, who had enjoyed good health, and who had all the appearances of strength of constitution, was seized, on the 2nd October, while in the street, with an apparent epilepsy. On the 3rd, he was carried to the Hotel Dieu, in general convulsions, with loss of sense. He was immediately bled. 4th. The intellectual functions completely restored—sensibility natural. Professor Recamier only prescribed a ptisan. 5th. The same—appeared remarkably well. In the night of the 5th, he had furious delirium. 6th. Monomaniac delirium, the patient being persuaded that he was poisoned, and that he had but a few hours to live. There was no fever—no disturbance of any of the visceral functions—judgment perfectly correct on every other subject. *Sinapisms to the feet—diluents.* Under the firm conviction that the attendants were employed to administer poison, he refused all drink, and suddenly and unexpectedly expired in the evening.

*Dissection.* Body in a state of embonpoint. In the head, the tunica arachnoidea, to a great extent, was opake and thickened. The meninges covering the cerebellum were also thickened and opake. The pia mater was strongly injected—the brain was rather less firm than natural, and on its surface were numerous red spots, which could not be removed by washing. The vessels of the tuber annulare were exceedingly injected. The lining membrane of the heart and large vessels was of a crimson colour. The mucous membrane of the stomach was somewhat softened towards the pyloric orifice, and reddened in those places. Some marks of phlogosis in the duodenum. No other morbid appearance.

*Remarks.* M. Martinet observes that, were it not for fear of extending this Memoir too far, he would here take an opportunity of stating a series of cases of an opposite kind, where patients had carried on a protracted existence, under organic lesions of great extent and intensity, and yet with far less disturbance of functions than in the above patients, where the organic lesions were comparatively trifling. Such cases, however, are very familiar to every pathologist. Indeed, it is manifest, that a person corporeally enfeebled by antecedent illness, is often more capable of bearing up under organic diseases than a robust person who is, for the first time, taken ill with an acute disease. Upon the whole, M. Martinet thinks himself justified in drawing the following conclusions.

"1mo. That the vital power or resistance differs in different individuals, and that the exterior aspect of the body, the constitution, and the temperament, are but feeble indices of this power—hence the fallacy of prognosis.

"2ndo. That it is by no means necessary that an inflammation should be intense in order to destroy life, if the power of resistance be of low rate in the individual.

"3tio. That death may take place without any appreciable mark of organic change being left, post mortem—while, in other cases, according to the degree of vital power—life may subsist under organic changes of the most profound and extensive kind.

"4to. That it is in the medical history of each individual (or in other words, an investigation of idiosyncrasy) that the physician will obtain the best data on which to ground his prognosis."

We are of opinion that there are many useful hints and just reasonings in M. Martinet's paper, and that the subject is one of very great interest to the medical practitioner.

---

2. *Resuscitation.** Our readers are aware that some discrepancy of opinion prevails, respecting the *power* which we possess of restoring suspended animation. If we are to believe the accounts transmitted to the Humane Society, there would seem to be scarcely any limits to this power, in respect to the period which elapses between suspension and restoration of the vital phenomena. Mr. Brodie and some others in this country, on the other hand, are very sceptical respecting the said power of resuscitation, after the cessation of the heart's action, which they suppose (from experiments on animals) cannot continue more than a few minutes after the respiratory function ceases. We confess that, from what we have actually seen of these resuscitations and attempts at resuscitation (which have been not a few) we are led to the same views as are entertained by Dr. Paris and Mr. Brodie. Our cotemporaries of the North are of a different opinion, and they have published an analysis of Dr. Roesler's thesis in support of their opinion. Of this analysis we shall present the prominent features, being desirous that the means of saving human life should neither be depreciated not rated too highly.

* Dr. Roesler's Inaugural Dissertation, Tubingen, 1824.—*Ed. Journal*, No. 82.

The first inquiry instituted by Dr. Roesler was to determine whether, in death by submersion, the air passages are really obstructed by mucus and water—a subject which has occasioned much controversy. The result of 45 experiments made by Dr. R. was, that in every instance a very small quantity of frothy mucus might be seen about the bifurcation of the trachea—but no water whatever—the quantity of mucus being wholly insufficient to produce the slightest obstacle to artificial inflation of the lungs. The attempt at washing out the mucus by means of warm water was not merely useless, but injurious. Not so the following:—

"This process was instituted, in order to determine the average number of recoveries by the inflation of temperate air, and the longest period of submersion after which animation might be restored,—with the view of afterwards comparing with these *data* the corresponding results obtained with heated air. The plan pursued was the following. As soon as the animal was removed from the water, it was carefully dried, laid in a warm, but well ventilated place, and rubbed, and finally swathed in flannel. The artificial inflation was then immediately begun, and was continued for half an hour or three quarters of an hour; after which, if no signs of resuscitation appeared, it was abandoned. At the same time, the frictions were frequently resumed, the body was kept well warmed, and as soon as any sign of returning life appeared, stimulants were introduced into the stomach. The only important particular which has been omitted in his description is the number of inflations; but as far as can be gathered from the details of the experiments, they appear to have been about 20 per minute." 209.

The average number of recoveries was 3 to 8. And next comes the great question as to the period of submersion. We have already stated the opinion of Mr. Brodie and Dr. Paris. It may be, that the latter author has thrown too much of an air of ridicule over a grave subject, by comparing the stories of Divers, and the reports from humane societies, with the ancient tales respecting "the Nymphs and Mermaids whose residence is in grottoes beneath the waves of the sea." But on the other hand, our northern cotemporaries are forced to admit "that the reports which have emanated from humane societies, and various other quarters, are too sanguine in their expectations of success in cases of long-continued submersion." The first train of experiments were three in number—two on rabbits and one on a cat. The periods of submersion were $5\frac{3}{4}$, $9\frac{1}{2}$ and $11\frac{1}{4}$ minutes, and in all the three cases, resuscitation was effected. But, *audi alteram partem.* "He several times failed when the animals were taken out of the water instantly after they seemed to have expired." It appears to us, therefore, that as the above three experiments were the only ones our cotemporaries could select out of 45, which were at all in support of their doctrines, they have very little indeed to boast of, granting that no fallacy crept in, or no colouring was given to the said experiments—fallacies and colourings which they themselves acknowledge to have often disfigured reports in this country, and which may (without prejudice be it spoken) occur in the reports of men in other climates also.

A part of the investigation relates to the relative value of cool and warm air in the treatment of suspended animation. The results appear favourable to the employment of heated air for the inflation of the lungs—" but enough has not been done to determine its precise value."

We have only one more observation to make on this subject, namely, that men are not cats or rabbits. Experiments on the inferior animals, will rarely apply to the human species. We have seen rabbits digest a good belly-full of parsley with both par vagums cut, and streams of electricity passing constantly along the nerves. But how would such an experiment succeed in man.

---

3. *Etiology of Goitre.** Dr. Drug, who practised some time at Matlock bath, Derbyshire, conceives that he has discovered the cause of goitrous affection—namely, *sour oat-cake*, aided by other innutritious diet, as inferior potatoes, &c. We apprehend that this is a very limited view of goitre and cretinism. We observe the former more abundant in some, than in other parts of Switzerland, though the diet is the same: for instance, in the valley of the Rhone we see hardly any thing else than cretins and goitres, while in the valley of Chamouny, separated only by the *Col de Balme* from the other valley, we see very few of either diseases. This contrast must arise, therefore, from something in the air or water, or both—for the food and mode of living are the same. Moreover, we trace bronchocele along the whole course of the Rhine, from Schaufhausen to Cologne, its frequency gradually and progressively decreasing as we descend that magnificent stream. What can this be owing to? For our own parts, after viewing with no inattentive eye, the moral and physical conditions of those inhabitants of the Alpine regions where cretinism and bronchocele abound, we have come to the conclusion that one of the main causes of bronchocele especially is to be found in the *water*, however much this cause may have been scouted by some medical and scientific travellers. We do not mean to say that it is the *snow-water* which has any thing to do with the complaint. But who can tell the thousand matters with which the Alpine waters are impregnated?—Every stream, great and small, that takes its origin from the glaciers, and other elevated crusts of ice or snow, becomes, in a short time, perfectly *white* with the particles which it wears from off every rock and mineral substance, in its noisy and precipitous route to the lakes below. Hence, a bottle of water taken from any of these streams, lets fall, on standing, a prodigious deposit of earthy and saline or mineral substances. When we consider the infinite variety of materials constituting the beds and banks of these Alpine streams, and see the quantity of *attritus* which they carry with them, and which are

---

* Dr. Thomas Drug. Med. and Phys. Journal, January, 1825.

swallowed by the inhabitants, we can have very little doubt that such ingredients have no trifling influence on the physical organization of man. This supposition is strengthened by the fact which we have just mentioned, that bronchocele gradually and progressively decreases, as we descend the Rhine—a river that rises in, and is chiefly supplied by, Alpine waters. It is hardly necessary to observe that, as the river increases its distance from the Alps, it lets fall the matters with which it was impregnated, as well as becomes mixed with auxiliary streams from countries not Alpine. This change is remarkable also in the Rhone. The upper Rhone, where it falls into the lake of Geneva, is turbid even to whiteness; but its waters, while nearly quiescent, in the lake, become clear, and pass through the city of Geneva like translucent streams of bluish crystal. Among those who inhabit the banks and drink the waters of the upper or turbid Rhone, (namely in the Vallais) there are twenty cretins and goitres, for one that can be seen on the banks of the lower or filtered Rhone, in its progress to the Mediterranean. Although we do not contend that *all* this difference is owing to the change in the waters; yet, taken in connexion with the Rhine, we think it forms a strong ground of presumption in favour of the goitrifactive (if we may use such expression) influence of Alpine waters. That the cause of bronchocele cannot be traced to sour bread, or indeed to any particular article of diet (the water excepted) at least in Switzerland, is proved by the fact, that English children (who live as well as people in England) cannot be kept long at Geneva, or in any other part of Switzerland, without having enlargements of the thyroid gland. This fact is well known to those English families who sojourn in that romantic country.

But it may be asked, can the waters in Derbyshire, or other goitrous countries in England, be the cause of bronchocele? We may answer, that it is much more probable that the cause of the disease should reside in the earth than in the air—and if in the *former*, surely the water which we drink is the most likely vehicle for its conveyance into the constitution. There is a mineral or saline substance in nature which is capable of removing the swelling of a thyroid gland—for instance iodine;—and why should there not be another saline or mineral substance which is capable of producing the said swelling?—The same valley which issues forth the miasma that causes ague, gives birth to the tree that furnishes the quinine, which cures the disease.

When on this subject, we may remark that, from an attentive survey of the Alpine valleys, it appeared to us that cretinism and bronchocele were only two prominent features of one great physical and intellectual deterioration which pervades the inhabitants of scenes the most romantic, sublime, and beautiful that human eye has ever yet beheld. It is not the swelled neck, the enormous head, and the vacuity of mind alone, that arrest our attention in traversing the Alpine regions. The whole corporeal fabric, with all its intellectual prerogatives, is stunted, deformed—and, as it were, aborted! Well might Goldsmith say—

"Man is the only plant that dwindles here."

Every feature and phenomenon of Nature around is wild, grand, and impressive—while he who is master of the whole, and the alleged image of his Creator, is an abomination to the sight.

